# Risk Factors, Prophylaxis, and Treatment of Venous Thromboembolism in Congenital Heart Disease Patients

**DOI:** 10.3389/fped.2017.00146

**Published:** 2017-06-19

**Authors:** Michael Silvey, Leonardo R. Brandão

**Affiliations:** ^1^Department of Pediatrics, Children’s Mercy Hospital, Kansas City, MO, United States; ^2^Department of Paediatrics, University of Toronto, Toronto, ON, Canada

**Keywords:** congenital heart disease, venous thromboembolism, venous thromboembolism prophylaxis, venous thromboembolism treatment, congenital heart disease and thrombosis, venous thromboembolism risk factors

## Abstract

Congenital heart disease (CHD) is a common condition in the pediatric population, affecting up to 1% of all live births (i.e., around 40,000 newborns/year in the United States). Although CHD does have a wide range of severity, by the age of 5 years approximately 80% of patients will require at least one surgical intervention to achieve a complete/palliative cardiac repair. Today, in light of their much-improved surgical survival, the care of these patients focuses on morbidity prevention and/or treatment. One such morbidity has been the increased frequency of thrombotic occlusions [e.g., cardioembolic arterial ischemic strokes; arterial, cardiac, and/or newly created shunt thrombosis; venous thromboembolism (VTE)]. Patients with CHD are at high risk of developing thrombosis due to the disruption of blood flow, CHD-related coagulopathy, inflammation, and/or platelet activation secondary to extracorporeal circulation support required during open-heart surgery or as a bridge to recovery, which can increase thrombus formation. In this article, we will discuss how the coagulation system is altered in patients with CHD in regard to the patient’s anatomy, procedures they undergo to correct their congenital heart defect, and other risk factors that may increase their thrombotic risk, focusing on VTE. We will also discuss the most recently published reports pertaining to guidelines on prophylaxis and treatment of VTE in this population. Finally, we will briefly address the long-term VTE outcomes for patients with CHD.

## Introduction

Congenital heart disease (CHD) is a common condition that affects approximately 40,000 births/year in the United States. Most cardiac birth defects classified as CHD occur in healthy children and are relatively minor (1). By contrast, until recently, complex CHD was accompanied by a significant mortality rate. Although survival has significantly improved, CHD patients are still at risk of significant morbidities particularly after their cardiac surgeries (2). Therefore, a better understanding of venous thromboembolism (VTE), one of the most common types of thrombotic occlusions occurring in this specific group, has become imperative, due to the increasing VTE incidence in hospitalized pediatric patients, including CHD patients (3–5). Moreover, it is well known that CHD is one of the main underlying conditions contributing to VTE in pediatric patients (6, 7).

Congenital heart disease patients are at high risk of developing thrombosis for various reasons: the type of cardiac defect, CHD-related complications, and stage of their care. For example, many of the structural cardiac abnormalities lead to changes in blood rheology or to natural anticoagulant deficiencies due to hepatic hypoperfusion secondary to impaired heart function, further contributing to a hypercoagulable state. In addition, many patients undergoing open-heart surgery necessitate cardiopulmonary bypass, where the required blood product exposure can lead to hemodilution and coagulation derangements. Finally, the increasing use of central venous catheterization in the care of such patients has also contributed to the VTE incidence escalation in this patient population.

This review will discuss several factors that increase the risk of developing thrombosis in CHD patients, including coagulation system changes due to their CHD and the procedures that they undergo to correct their heart defects. VTE prophylaxis and treatment will also be discussed. As these patients now have a normal or near normal lifespan, long-term VTE outcomes will also be discussed briefly.

## Differences in the Coagulation System of CHD Patients

The coagulation system in neonates and infants is physiologically distinct from adulthood. Neonates and infants have lower concentrations of both pro-coagulant and natural anticoagulant proteins compared to adult values, as a reflection of the physiologic developmental hemostasis process that occurs in all children. Circulating levels can take up to 12 months, and in some instances only reach adult levels in adolescence (8, 9). Furthermore, neonatal and infant CHD patients have lower concentrations of pro-coagulant and natural anticoagulant proteins than normal age-appropriate controls (10–13). The cause for lower concentrations of coagulation proteins is believed to be multifactorial (i.e., increased consumption, decreased production, and increased fibrinolysis).

As CHD patients have their surgical corrective procedures, the coagulation profile showing decreased pro-coagulant and anticoagulation proteins is enforced. Odegard et al. examined coagulation profiles of 37 patients with hypoplastic left heart syndrome before their stage I palliation, bidirectional Glenn, and before and after the Fontan procedure. The investigators found that all coagulation proteins were at significantly lower concentrations except for factor VIII, which only increased after the Fontan procedure (12). The authors posited that the increased factor VIII production was due to increased production from liver sinusoidal epithelium secondary to chronically elevated central and hepatic venous pressures associated with Fontan physiology.

Congenital heart disease patients may also present with thrombocytopenia (10), which is multifactorial. For instance, thrombocytopenia can be secondary to hypoxic inhibition of platelet production, increased platelet destruction with shunt placement in single-ventricle physiology, or increased RBC production. Lill et al. hypothesized that right-to-left shunts in CHD patients decreased megakaryocyte delivery to the lungs, impairing platelet production in the pulmonary bed (14). In addition, CHD patients have been shown to have abnormal platelet function. Maurer et al. reported decreased aggregation when platelets were exposed to platelet agonists (15). Furthermore, Bailly et al. found patients with systolic flow abnormalities to have abnormal platelet function (16), which could be explained by the fact that CHD patients may develop acquired von Willebrand disease, known to affect platelet aggregation testing (17). Finally, CHD associated with other genetic disorders may also present with platelet dysfunction. CHD patients with Noonan’s syndrome, velocardiofacial syndrome, and Jacobson’s syndrome can have quantitative or qualitative platelet defects, which can increase risk of bleeding.

## The Coagulation System and Cardiopulmonary Bypass (CPB)

Patients who undergo CPB are temporarily exposed to a non-physiologic circulatory system, which disrupts their coagulation system. Once CPB is initiated, the contact factors are activated, and tissue factor is exposed, which lead to increased thrombin generation. Upon thrombin generation, production of fibrin as well as protein C activation (18) ensues. Therefore, as a result of the increased thrombin generation and subsequent fibrin deposition within the CPB circuit, anticoagulation is required during CPB to prevent excessive thrombus formation. In addition to consumption of several coagulation factors within the circuit, platelets are significantly affected during CPB. When CHD patients are placed on CPB, platelet activation occurs immediately (19, 20) followed by platelet consumption, leading to an acquired quantitative or qualitative platelet defect (21). Patients younger than 1 year of age are more likely to have this complication. Second, hemodilution due to priming of the CPB circuit can also lead to thrombocytopenia. Third, CPB confers a pro-inflammatory state as it activates both humoral and cellular aspects of the immune system (18). With the activation of the inflammatory response, endothelial cells will express tissue factor, further promoting thrombin generation (22). Consequently, in light of the CPB-induced coagulopathy that may develop, CHD patients have an increased risk of bleeding in the immediate post-operative period and can be treated with blood-derived products, anti-fibrinolytics, and hemostatic agents such as recombinant activated factor VII (23), as needed.

## Cyanotic CHD and Thrombosis Risk Factors

Patients with cyanotic CHD (e.g., Tetralogy of Fallot, tricuspid atresia, and single-ventricle physiology) can be at high risk of developing VTE due to the surgical procedures required to correct their originally abnormal cardiac anatomy. The Blalock–Taussig (BT) shunt is a common surgical procedure in neonates with single-ventricle physiology, where a shunt is created between the subclavian artery and the ipsilateral pulmonary artery to increase pulmonary blood flow (24, 25). This shunt creates a low blood flow area that increases the risk of thrombosis, and surgically removed shunts have been found to be obstructed. Of note, complete BT shunt obstruction creates a significant decrease in pulmonary blood flow, almost invariably requiring an immediate mechanical thrombectomy. The frequency of BT shunt thrombosis reported in the literature ranges from 1 to 17% (24). Wells et al. reported BT shunt histopathology for the detection of shunt obstruction from 155 patients and found a median shunt lumen narrowing of 34%. Moreover, 21% of cases showed a shunt stenosis greater than 50%. The identified risk factors for obstruction were smaller shunt size, age less than 14 days, and shunt placement while on bypass (26).

A second palliative surgical procedure to increase oxygenation in patients with single-ventricle physiology is the bidirectional Glenn procedure. This procedure connects the superior vena cava to the right pulmonary artery (27). The largest concern regarding thrombosis is the development of pulmonary embolism, with a subsequent increase in pulmonary vascular resistance, making patients unsuitable for further palliative surgeries (25). Though Glenn Shunt thrombosis is rare, a study by Manlhiot et al. comprising 203 cardiac operations performed in single-ventricle patients showed that the second highest occurrence of VTE (12%) corresponded to cavopulmonary shunt operations (28).

The final palliative surgical stage for single-ventricle physiology patients is the Fontan procedure. This procedure involves rerouting the systemic venous return directly to the pulmonary arteries (29). The Fontan procedure has undergone many modifications, though the mechanism of the anastomosis of the inferior vena cava to the pulmonary arteries has remained unchanged (25). Patients undergoing the Fontan procedure are also at an increased risk of developing thromboembolism, and the reported incidence of thromboembolism ranges from 3 to 20% (30). Identified post-Fontan thrombosis risk factors include passive blood flow, chronic venous hypertension, and atrial arrhythmias. Patients undergoing Fontan procedures were found to have elevated factor VIII levels, which may further increase VTE risk (12). Patients may or may not have a fenestration placed within the atria; the latter procedure could lead to the development of paradoxical emboli if a thrombotic source is present within the right cardiac chambers. As many modifications have been made to the Fontan, Coon et al. examined whether thrombosis rates and risk factors varied among patients undergoing different types of procedures. No difference was found in thrombosis incidence in either the pulmonary or venous system in any of the modifications (31). These findings suggest that the physiology of the Fontan circulation leads to thrombus formation.

Additional cyanotic CHD conditions do not pose higher thrombotic risk. The higher risk of these patients developing thrombosis is more strongly associated with CPB and the coagulation abnormalities described earlier.

## Acyanotic CHD

Patients with acyanotic congenital heart lesions do not usually have a significant rate of thrombotic complications. Depending on the type of atrial/ventricular septal defect, the patient may or may not undergo CPB surgery to close the defect (32). Ventricular septal defects are more likely to require surgical closure than atrial septal defects. Recent advances in interventional closure devices, specifically for perimembranous ventricular septal defects, have decreased the need for CPB (33, 34).

## Cardiac Catheterization

Congenital heart disease patients commonly undergo a cardiac catheterization procedure for both diagnostic and treatment purposes. When undergoing cardiac catheterization, the femoral artery or femoral vein is accessed for catheter insertion immediately prior to a bolus of unfractionated heparin (UFH). Thrombosis and pulse loss after catheterization are known complications of this procedure. Venous thrombosis has a prevalence ranging from 0 to 27% and the reported prevalence for femoral artery occlusion is between 0.6 and 9.6% (35–38). Post-cath thrombosis risk factors in both the femoral artery and vein include younger age, patient size, use of larger catheters, longer procedural time, and need for repeat procedures.

## VTE Prophylaxis for CHD Patients

Due to the high risk of developing thrombosis with CHD and cardiac surgery, studies have examined the role of thromboprophylaxis to decrease thrombosis development. Low molecular weight heparin (LMWH), oral vitamin K antagonists (OVKA; i.e., warfarin), and aspirin have been successfully used as primary thromboprophylaxis options for CHD patients undergoing cardiac procedures; this strategy has also decreased patient mortality (39, 40). To help clinicians decide which prophylaxis is more appropriate, evidence-based guidelines for the management of CHD patients after cardiac surgery have been published (41–43).

### Antiplatelet Therapy

Aspirin is widely used; commonly, it is typically started on patients after systemic to pulmonary shunt placement as long-term prophylaxis. Additionally, aspirin may also be considered for primary prophylaxis in children undergoing the Fontan procedure (44). The usual aspirin dosing for prophylaxis is 3–5 mg/kg daily, and treatment duration can vary. Aspirin prophylaxis may be used in patients undergoing cardiac catheterization if a device is placed during the procedure. Clopidogrel, another antiplatelet agent, is increasingly being used in children (45). Despite its widespread use, a recent trial showed clopidogrel was not more effective than placebo in reducing mortality or shunt-related morbidity in high risk CHD patients (46).

### Anticoagulation Therapy

Unfractionated heparin and warfarin are the most commonly used anticoagulants for patients undergoing cardiac surgery. UFH is used mostly as an immediate post-operative medication for thromboprophylaxis, particularly in patients undergoing a systemic to pulmonary shunt placement. For patients with clinically significant post-operative bleeding, low-dose standard heparin may be preferred prior to starting antiplatelet therapy. Systemic heparinization may also be continued with additional antiplatelet therapy if there is sustained high risk of developing thrombosis. In such cases, further evaluation regarding the thrombosis risk will determine whether the UFH infusion needs to be switched to LMWH in addition to aspirin.

Warfarin prophylaxis may be started on patients after the Fontan procedure for, at least, 3–12 months. Warfarin may be continued for much longer periods when in the presence of certain thrombosis risk factors. Patients are usually dosed to maintain an international normalized ratio (INR) between 2.0 and 3.0 to minimize bleeding risk. Point-of-care monitoring is likely an option for outpatient INR laboratory monitoring in children receiving long-term oral anticoagulation with an OVKA (47). Direct oral anticoagulants (DOACs) are not approved for use in the pediatric population and are only recommended for pediatric patients if they are participating in a clinical trial. At present, there is only one clinical trial open for the use of a DOAC in CHD patients (Apixaban in Children with Cardiac Conditions; NCT02981472).

Patients undergoing a cardiac catheterization usually receive a heparin bolus prior to and possibly during the catheterization, if the procedure extends beyond 2 h. Patients are usually given 100 U/kg prior to the procedure, but smaller doses have also been employed (48). Activated clotting time allows monitoring monitor anticoagulation, and heparin bolus dosing should be determined following the American Heart Association Guidelines (43).

## Risk Factors of VTE in CHD Patients

Besides cardiac procedures, additional thrombosis risk factors have been identified in CHD patients (Table 1). Likely, the most pervasive VTE risk factor in children is central line (CVC) use. Central venous access is vital to manage children post-operatively, and many children with CHD will have multiple CVCs placed at once in both the lower and upper extremities. Moore et al. prospectively evaluated CVC-related VTE in CHD patients *via* lineograms at CVC removal and reported a VTE prevalence of 20%. All lines were placed in the jugular veins; 60% of thromboses were located in the jugular veins while the remaining 40% were located in the right atrium (49). Additionally, Hanson et al. found 41% of CHD patients developing a VTE to have a CVC-related event. The time length of CVC placement was directly associated with thrombosis (50).

**Table 1:** Published literature regarding thrombosis risk factors and outcomes in CHD patients.

Reference	Study design	Prospective/retrospective	Number of patients	Main VTE findings	Limitations
Faraoni (51)	Database review of CHD patients	Retrospective	27,492	Thrombotic complications in 3.9% of surgical CHD patients; younger age, single-ventricle physiology associated with increased risk	Retrospective; no access to pt charts, data could be missed or miscoded, causality not established

Manlhiot et al. (28)	Cohort of CHD patients who developed thrombosis and outcomes	Retrospective	192	Serious complications occurred with 17–24% of thrombi, 13% of patients had bleeding complications	Retrospective, no standardized follow-up, unable to assess for thrombophilia, asymptomatic thrombus not identified

Jensen et al. (52)	Cross-sectional study examining pulmonary and cerebral thrombosis	Prospective	98	Prevalence of cerebral and pulmonary thrombosis: 47 and 31%	Unable to determine when the thrombotic event occurred, small sample size

Emani et al. (53)	Single-ventricle physiology cohort	Retrospective	512	51 patients developed thrombosis (10%); patients with thrombosis had longer ICU and hospital stays, single-ventricle physiology and CBP were risk factors	Retrospective, small number of patients, small number of thrombotic events

Wessel et al. (46)	Multicenter, randomized double-blind, placebo controlled study examining clopidogrel in CHD patients	Prospective	906	Clopidogrel did not reduce mortality or shunt-related morbidity	Heterogeneity of the patient population, could not compare Aspirin + clopidogrel vs. clopidogrel alone, difficulty to diagnose shunt thrombosis

Idorn (54)	Population-based study of Fontan patients	Retrospective for prevalence, prospective for laboratory testing	210	Thromboembolic prevalence was 8.1%, no evidence of hypercoagulability in the patient groups	Blood samples not taken at the time of thrombotic event, could not establish reference ranges for coagulation tests

Manlhiot (39)	Cross-sectional study of patients undergoing cardiac surgery	Retrospective	357	Thrombotic incidence was 40 and 28% after initial palliation and superior cavopulmonary connection, thromboprophylaxis with enoxaparin was associated with a reduced risk of thrombotic complications, thrombosis was associated with increased mortality	Retrospective, no control group

Hanson et al. (50)	Observational study for VTE risk factors in critically ill CHD patients	Prospective	1,070	VTE incidence was 3.8% with 37% of VTEs being CVC-related; VTE was associated with single-ventricle physiology, and more CVC days	Heterogeneity of the patient population, no screening of VTE

Tzanetos et al. (55)	Observational study for predictors of thrombosis in single-ventricle physiology patients	Prospective	16	Perioperative thrombus incidence was 31%, thrombus associated with longer CPB times, poor ventricular function, and lower antithrombin and tissue plasminogen activator antigen levels	Small sample size, no control group, no long-term follow-up data, unequal distribution of patients among surgical intervention groups

Monagle et al. (44)	Multicenter, randomized trial comparing ASA vs. heparin/warfarin for primary prophylaxis	Prospective	111	No difference between the ASA vs. heparin/warfarin arms	Poor recruitment leading to less than optimal number of patients

Manlhiot et al. (5)	Single center, cohort study of CHD patients	Retrospective	1,361	VTE incidence was 11%, VTE associated with increased number of ICU and hospital days, cardiac arrest, and higher mortality	Retrospective, possible incomplete medical records, unable to prove causality between VTE and morbidity/mortality

Kim (56)	Cohort study of CHD patients	Retrospective	200	VTE occurred in 13 patients (6.5%), mostly happening within 1 year after the Fontan procedure	Retrospective, small sample size

Hanslik (57)	Cohort study of CHD patients	Prospective	90	VTE was detected in 25 patients (28%), all VTEs occurred in the right jugular vein	Possible overestimation of sensitivity of diagnostic tests, follow-up monitoring not done

Li et al. (40)	Multicenter non-randomized, observational study examining aspirin efficacy in lowering risk of death and shunt thrombosis	Prospective	1,004	Patients on ASA with lower risk of shunt thrombosis and death	Non-randomized, observational, no other antiplatelet or anticoagulation given, shunt characteristics not related to shunt thrombosis

Cholette (58)	Observational study of neonates undergoing cardiac surgery	Prospective	22	5/22 (23%) patients had evidence of thrombosis, C-RP elevation was the only predictor of thrombosis development	Small sample size

Kaulitz (59)	Cohort of patients undergoing total cavopulmonary anastomosis	Retrospective	142	10 patients (7%) suffered thrombotic events, 8 patients with VTE, and 2 patients with stroke	Retrospective, small number of patients

Gurgey (60)	Review of CHD patients with thrombosis and thrombophilic factors	Retrospective	28	Overall frequency of Factor V Leiden and prothrombin Gene mutation was 22%, 5 patients had the FVL gene mutation and 1 pt had the prothrombin gene mutation	Retrospective, no control group, small sample size

Seipelt (61)	Cohort study of CHD patients	Retrospective	101	Thrombotic events happened in 13/85 hospital survivors (15.3%)	Retrospective, small number of patients

Jacobs (62)	Survey of patients who underwent Fontan procedure and aspirin use	Retrospective	72	No thrombotic event was documented (0%)	Retrospective, no control group, small number of patients

Coon et al. (31)	Echocardiogram findings in Fontan patients	Retrospective	592	Thrombosis prevalence 8.8%	Retrospective, various time intervals until imaging was performed

Petaja et al. (63, 64)	Single center, cohort study of CHD patients	Retrospective	1,499	Central venous thrombosis incidence was 1.1%; mortality associated with central venous thrombosis was 40%	Retrospective

Petaja et al. (63, 64)	Single center cohort study of CHD patients	Retrospective	10	3 patients who developed thrombosis had decreased antithrombin or protein C levels and elevated plasminogen activator inhibitor levels	Retrospective, small sample size

Rosenthal (65)	Cohort study of patients undergoing the Fontan procedure	Retrospective	70	14 patients (20%) developed thromboembolism with 12/14 thrombi located in the venous circulation	Retrospective, small number of patients

*CHD, congenital heart disease; VTE, venous thromboembolism; ASA, aspirin; ICU, intensive care unit; CPB, cardiopulmonary bypass; C-RP, C-reactive protein; CVC, central venous catheter*.

Other studies have examined additional potential VTE risk predictors, including clinical and laboratory markers. Tzanetos et al. examined 19 patients who underwent palliative surgery for single-ventricle physiology and found that those who developed thrombus had worse pre-operative ventricular function, longer CPB time, decreased circulating antithrombin levels, and increased tissue plasminogen activator antigen concentrations (55). Similarly, Petaja et al. examined 10 neonates who underwent CPB. Three patients developed thrombosis and were found to have decreased circulating antithrombin or protein C levels and elevated plasminogen activator inhibitor levels (63).

Aspirin prophylaxis response has also been explored as a predictor of VTE development. Emani et al. described decreased aspirin response as a predictor for VTE development; 95 patients were followed 30 days after initiation of aspirin therapy. Patients not responding to aspirin, particularly patients weighing less than 5 kg had a higher thrombosis rate compared to those showing adequate aspirin response (53). Mir et al. examined the incidence of aspirin responsiveness in 20 infants with single-ventricle physiology after palliative heart surgery by measuring urine thromboxane (UTX) levels and thromboelastography (TEG). The authors found aspirin resistance in around 80% of infants using TEG; no patients had adequate UTX levels and clinical variables such as age, weight, hemoglobin level, and platelet count were not associated with aspirin resistant status (66).

Congenital heart disease patients who undergo cardiac surgery are also at risk for developing ischemic stroke. Domi et al. examined 5,526 patients who underwent cardiac surgery and found that the incidence of arterial ischemic stroke/cerebral sinus venous thrombosis was 5.4 strokes per 1,000 children. Risk factors associated with stroke included older age, longer duration of CPB, reoperation, and number of days hospitalized after the operation (67).

## VTE Treatment for CHD Patients

Patients with an acute, symptomatic VTE and no relevant contraindication are treated with anticoagulation, namely, systemic UFH or LMWH (41–43). The anticoagulation modality of choice depends on the circumstances of the patient (surgical needs, liver/kidney dysfunction). Patients started on systemic anticoagulation for VTE are usually treated for 3–6 months (41–43). Moreover, patients receiving systemic anticoagulation should be monitored throughout treatment for bleeding complications, and frequent laboratory testing is recommended to ensure their anticoagulation dosing is therapeutic. For patients undergoing UFH therapy, a target PTT of 70–100 s, which correlates to an anti-factor Xa level of 0.3–0.7, is considered therapeutic. For patients undergoing LMWH therapy, an anti-factor Xa level of 0.5–1.0 is considered therapeutic. Patients may be transitioned to warfarin therapy depending on their clinical circumstances, and the target INR for these patients is 2.0–3.0. If the patient does have a life or limb-threatening thrombus, thrombolysis should be strongly considered (41–43).

## Outcomes for CHD Patients with VTE

Patients who undergo CPB surgery can be at significant risk of developing comorbidities if they develop VTE (Table 1). Manlhiot et al. retrospectively reviewed 1,361 CHD patients after cardiac surgery and described morbidities associated with VTE. VTE was associated with increased number of ICU days and increased length of stay. VTE was also associated with increased risk of cardiac arrest, and thrombosis-related mortality was 5%. However, in light of the retrospective study design, VTE causality could not be inferred (5). Pejata et al. also conducted a single center retrospective review to evaluate mortality in 20 CHD patients after developing thrombosis. VTE was associated with increased mortality rate, as VTE patients had a 40% mortality rate compared to patients who did not develop VTE (8.3%). Again, due to the study limitations, causality between VTE and mortality could not be established (64).

## Long-Term Outcomes for CHD Patients

Post-thrombotic syndrome (PTS; chronic venous insufficiency secondary to vessel damage) is recognized as an important long-term complication in CHD patients. Brandao et al. examined 70 CHD patients aged 0.5–5 years after their surgical repair and found that 30% of patients had clinical evidence of PTS (68). Most patients had lower limb PTS, and all patients had mild PTS.

Post-thrombotic syndrome after cardiac catheterization is a newly recognized long-term complication for patients. Luceri et al. studied 62 patients who underwent cardiac catheterization and found that 40 children had PTS (prevalence 64%, 95% CI 51.3–76.3%). Most cases were mild, though seven children had clinically significant PTS (69).

Importantly, CHD patients seem to carry their increased thrombotic risk into adulthood. Jensen et al. reported a prevalence of cerebral and pulmonary thrombosis of 47 and 31%, respectively, in adult survivors (52).

## Conclusion

The risk of thrombosis development in CHD patients is multifactorial. Thrombotic events lead to short- and long-term complications for these patients. Although guidelines for thromboprophylaxis and treatment are currently available, more research is needed to increase the knowledge of how thrombosis develops in these patients and how to prevent this common complication.

## Author Contributions

MS performed the literature search and wrote the manuscript. LB also performed the literature search and edited the manuscript.

## Conflict of Interest Statement

The authors declare that the research was conducted in the absence of any commercial or financial relationships that could be construed as a potential conflict of interest.
